# The Apparel Destined by Nature for the Absorption of the Roots of the Temporary Teeth

**Published:** 1844-06

**Authors:** 


### ARTICLE IX.

*The Apparel destined by Nature for the Absorption of the Roots of the Temporary Teeth.* Translated from the French of Delabarre. By * A. *

If the teeth of man grew as their possessors gradually increased, like what is observed in some species of fish, a second dentition would have been necessary; but this is not the case; the temporary teeth having acquired their dimensions, do not continue to increase any longer, whilst, on the contrary, the jaws of the fœtus are extended by reason of the general development of the individual. These provisionary teeth, that have at first lain obliquely in the fœtal jaws, which then were ranged so agreeably to the alveolar edges of the infant, and which, until the second or third year, are placed very near each other, commence about this period to become perfectly separated. This result of the increase of the maxillary circle soon calls for stronger teeth—teeth that may afford less fragile means to perform mastication. Consequently the teeth that have been silently growing behind the primitive ones, gradually announce their presence by moving there, which finally yield and give place to them. This singular phenomenon, at first unknown, has been explained in various ways; and the moulting of the teeth is still, to those who see only mechanical causes, a problem that they cannot solve. Formerly, very celebrated anatomists wrote, that the milk teeth were without roots: this error has been shared and repeated by their cotemporary dentists, who, probably, did not think proper to assure themselves of the fact. Yet the simplest dissection of a child's jaw would have dispensed with these useless commentaries. It is not more than fifty years since this question was decided, and the truth known; but where it still remained to be resolved, it was remarked by some, if the milk teeth have roots, how happens it, that, when they fall out, we do not find them? Instead of making anatomical researches, they formerly conjectured that the root, just as the horn of the stag, falls from the head that bore it, and that then the root gave birth to a new tooth.

Bunon, having made several preparations to assist him to understand the subject, and having acknowledged that the temporary and permanent teeth were perfectly independent of each other, endeavors to explain this destruction of the roots of the teeth.

This author supposed that the heat of the parts increased the action of second dentition; and the pressure which he thinks must be exerted by the crown of the permanent tooth upon the root of the temporary, were the causes of its destruction; he moreover conjectures, that it was either entirely consumed by the heat of the parts, or carried away by the saliva.

Fauchard combats this opinion, not believing, with reason, in this mechanical pressure, but supposing that this destruction results from a proper disposition of the juices of the temporary root, or from the action of the surrounding liquors.*

L'Ecluse, in 1764, pretended that the dental vessels do not any longer convey the juices to the milk teeth, whenever those of replacement have attained a certain degree of development; and hence, according to this writer, it follows that these small roots are broken by a sort of maceration.

Jourdain attributes the destruction of the roots of these teeth, and of the inter-alveolar partition, to several causes: 1st. To the pressure which he supposes to be exerted by the tooth of replacement. 2d. From the mechanism he adopts, he admits, with Fauchard, that the osseous juices which are carried to the milk teeth by *particular vessels,* overflow in the temporary teeth, acquire then a certain acrimony, changing its color, altering its substance, disposing it to decomposition as if it were injected with an acid, and that, finally, the residium of this dissolution disappears without any one's knowing by what means.

All these explanations could not convince Bourdet, who believes that the destruction of the temporary roots could not be effected by the means mentioned by these authors; and he was the first to observe that, at the time of moulting a temporary tooth, we

* Bichat has shared this opinion.

always find behind it a carneous tubercle which served as a sort of niter medial between the crown of the tooth of replacement and the root of the tooth to be replaced, so that all immediate contact was impossible. He thence concludes that this small tubercle exudes an acid juice, with the property of acting upon the temporary root that is nearest it. M. Laforque, having also remarked this small fungoid body, and supposing it to possess the function assigned it by Bourdet, designates it by the name of absorbing apparel. This, in effect, is to suppose a carneous tubercle being gradually developed before the tooth, whose progression it precedes, that continually augments in volume, and appears to consume not only the temporary root, but every thing that would oppose the march of the tooth; if it is not, what other organ is charged to do this?

Surely the increase of the osseous channel, that I have called the *Inter Dentis*, [alveolo-dental canal,] and the destruction of parts so compact as the temporary teeth and the maxillary bones, can only be effected by one of those phenomena placed under the influence of life.

We have shown, in a preceding chapter,* that the disposition of the crowns of the teeth within the alveoli is such, that there is never any contact between them and the surrounding parts, and that, consequently, there is no pressure. There is not, moreover, any arterial pulsation capable of destroying the neighboring organs, such as sometimes happens in the vicinity of large vessels, or of aneurism. We cannot, then, admit that the destruction of the temporary roots can only result from a friction of which we have demonstrated the utter impossibility.

We cannot, moreover, believe in the exuding of stagnant acid juice, for the presence of such a chemical agent would not only act upon the tooth that should be destroyed, but would also most indubitably cause inflammation, followed by suppuration and caries. Nature, always so provident, is bound to provide against such accidents; she sometimes, it is true, envelopes her simplest operations in a veil that at first appears mysterious, but which is soon drawn aside by physiological discoveries and exact research.

* The author here refers to another part of the work, from which this translation is made.—*Balt. Ed.*

On the subject of the destruction of the temporary roots, Hunter, in his Natural History of the Teeth, thus explains himself:—"It is very natural to suppose that this [wasting] was owing to a constant pressure from the rising teeth against the fangs or sockets of the first set; but it is not so, for the new alveoli raise with the new teeth, and the old alveoli decay in proportion as the fangs of the old teeth decay, and when the first set falls out, the succeeding teeth are so far from having destroyed, by their pressure, the parts against which they might be supposed to push, that they are still enclosed and covered by a complete bony socket. From this we see the change is not produced by a mechanical pressure, but is a particular process in the animal economy."

Fox believes that the destruction of the temporary teeth results from a pressure that is exerted by the permanent tooth upon the alveolus, and upon the root of the tooth that should yield it place. The mechanism adopted by this author, though not the true one, is still very ingenious. We will see what he says upon the subject:

"It has been observed that the pulps of the new teeth are placed behind the temporary ones, and in that situation they are very much crowded, and occupy but a small space. Now it is evident that as they advance in growth, they will require an increase of room, to obtain which, they must come forward, so as to form a larger circle."

"This effort first produces a considerable pressure against the bony partition placed between the temporary and permanent teeth, and upon the posterior part of the fangs of the shedding teeth. The pressure in this instance acts precisely in the sam e manner as it generally does in other cases where it is applied. It induces an absorption of the parts pressed against; and as the new teeth augment, the fore part of the socket which was formed around the pulp, and separated it from the temporary tooth, is removed by the process of absorption."

Thus we see this fungiform body has not been observed by Fox, and that his system, ingenious as it is, must of itself fall to pieces, when we carefully dissect the jaws of an infant of six or more years. Has M. Serres solved the problem in his *Nauvelle Theorie de la Dentition?* This author has promised to explain how the roots of the milk teeth are *worn away*, and he thus acquits himself: "The osseous interculary is opened from the bottom to the top by a *usure**—a slow destruction, resulting from a primordal law, of which no one can assign the natural cause."

This explanation is almost literally translated from Hunter. A little further on, he adds: "The roots of the temporary teeth are worn away by the same law that destroys the alveolar partition." Finally, he supposes a vacuum is formed by the destruction of the temporary root.

The science has gained nothing from these plausible hypotheses. If M. Serres had given himself the trouble to read the works of Bourdet and of M. M. Duval and Laforque, he would have seen that this law has established an organ, whose existence and functions have been conjectured by these learned dentists.

It is certain that there is not only a law, but also an agent charged by it to effect the destruction of every thing that would form an obstacle to *odontocie*, (dentition;) this agent would not have been so long unknown, if physiologists, instead of employing themselves in vain reasonings, had sought to interrogate nature about the fact. For scarcely has first *odontocie* been accomplished, when second dentition, at once, prepares all its forces to destroy that under the cover of which it is developed. While the crown of the tooth of replacement is only in formation, the exterior membrane of the matrice is simply crossed by some blood vessels; but as soon as it is completed, the capillaries are then developed in a very peculiar manner, and from a tissue as fine as cobweb; from this time the internal membrane, instead of continuing to be very delicate and of a pale red color, increases in thickness, and assumes a redder hue.

As was before said, it is at the instant in which commences the reaction of the coats of the matrice, that are conveyed from the gum to the neck of the tooth, that the plaiting of the vessels that enter into their tissue, compose a body of a carneous appearance, whose absorbents extend their empire over all the surrounding parts; it is, therefore, the dental matrice itself, that, after being dilated to serve as a protecting envelope to the tooth, is contracted not only to form this bud-like body which we find

* Waring.

immediately below the milk tooth, at the instant in which it naturally falls out, and whose volume is necessarily augmented as odontocie gradually goes on; but also a carneous mass by which the whole crown is surrounded, whose thickness is the more remarkable as the organ that it envelopes is nearer its orifice.

Is there, then, a dissolving fluid exhaled from this, that acts chemically on the surrounding parts, or do the absorbents, without any intermedial, destroy every thing that would obstruct the shooting up of the tooth? Not possessing positive proof, suitable to guide me in the decision of this question, and finding those of others of little importance, I will not attempt to answer them.

The vessels of the temporary tooth frequently remain entire, in the midst of the destroying apparel by which they are surrounded. They continue to convey the juices to the central part of the tooth, whilst the calcareous phosphate and gelatin have been taken away from round about them. Sometimes they are found strangled or even destroyed.

The natural moulting of the temporary teeth generally happens when almost all the root has been destroyed, but frequently not until the interior of the crown is hollowed out in the form of a capsule; nevertheless, diseases of the gums may determine its moulting, without there ever having been any absorption. Thus, whether the absorbents of the fungiform tubercle, by a sort of suction, pump the calcareous phosphate and gelatin from the temporary root, without any previous disposition, or whether an exhaled fluid effects this decomposition, they are both carried back in to the general torrent of the circulation, conformably to the law of vital decomposition.*

The vessels charged with this operation are more decided in the place that borders on the temporary root. When they are very red, as if inflamed, life, in fine, strikes with reiterated strokes, employs all its energy, and destroys every thing that forms an obstacle to the passage of the organ that replaces the one whose destruction has been determined on.

Force for the performance of certain phenomena, increases in

* Recent experiments made by Dr. Magendie at Paris, and M. Mayer at Beme, prove that absorption is peculiar to the veins, and render doubtful, according to these authors, that of the lymphatics.

the ratio of the resistance; hence the absorbing body is more decided as the parts to be destroyed are thicker or more solid; for the same reason, too, it is found to be very powerful behind a milk molar, that is about to be moulted, and whose strong roots have been entirely consumed. There is not, therefore, any vacuum occasioned by the destruction of the deciduous root, as has been gratuitously supposed by a modern author, for the volume of the apparel is in direct ratio to the destruction effected, and the progress of the tooth. Nor is there any of the *detruitus* remaining in the alveolus as Bunon remarks. The root of the tooth is absorbed in the same manner as all the parts that are touched either by the fungi-form tubercle that proceeds, or the body of the matrice itself.

There cannot be any abrasion of the temporary root by the crown of the permanent tooth, since there is no friction, this last being separated by the matrix from every thing that environs it, even until it has passed the aperture of the *Iter Dentis.* As soon as the crown of the temporary tooth falls out, we perceive that the permanent, which does not delay to be elevated to the same level as that which has preceded it. The dental matrix having passed through different states, and having fulfilled the functions that were assigned it, is effaced and leaves no other trace of its past existence than those two small eminences that delineate the festoons of the gums. If there is no room for the immediate action of the absorbing apparel upon the temporary root, which happens either when the teeth of replacement has not followed the *Iter Dentis,* or when the child is feeble, its destruction is not effected, and it may remain in its place during the whole period of life, though the permanent tooth sprout up not far from it.

The absorption of the root may sometimes occur, although the permanent tooth is still at a distance from it, but then the action that is performed, has wholly preceded *odontocie;* this is a new proof that it is not any mechanical action that determines the moulting of the teeth. Hunter and Fox have also noticed this fact. Thus the latter has avowed his embarrassment in explaining absorption as the result of pressure.

There is in my possession a curious piece of anatomy, it consists of a large second molaris of the upper jaw, between whose roots a *dens sapientiæ* is developed. The destruction of the roots has been effected in the same way as they are in the milk teeth; the *Iter Dentis* passes between them. This very rare fact proves that the annihilation of the roots of the teeth, may occur not only in the temporary, but also, all that are submitted to the action of the *absorbing apparel.* Thus it sometimes happens, that a temporary tooth, situated near that which should be replaced, is found to be partially destroyed by the fungi-form body destined to destroy its neighbor; so that the two milk teeth drop out together to be replaced by only one; an irregularity in the adult teeth may result from this, for when the other tooth is about to sprout out, finding its place occupied, it oftentimes deviates to the interior or exterior side.

If the development of the apparel is imperfect, or if the appendage of the sac has been destroyed by an injudicious operation, the tooth frequently remains imbedded in the alveolus and does not sprout. I have now in my possession the jaws of an adult, in the substance of which the *dens sapientiæ* still continue enclosed.

Immediate contact between the temporary and tooth of replacement cannot exist, when the last is enclosed in the maxillary bone, but it very frequently occurs, while it is encased in the exterior aperture of the *Iter Dentis*, when the tooth that should have been moulted, is found disordered, and in such a case the destruction of the temporary root is found imperfect, which would not be, were the mere contact the cause of the *usure* or absorption.

M. Duval, always so judicious an observer, has also made similar remarks.

I have seen many cases in which a last molar being found directed towards its neighbor, after their formation, has determined a sensible depression on the crown of this tooth, but its enamel was not at all altered, and its thickness was the same as all its other parts.

There nevertheless is a method of absorption that depends on a diseased affection, which is frequently met with in the practice of surgery; as when a bullet having remained several years in the thickness of the cellular tissue, presents numerous proofs that the capillaries are found to alter their economy. I also know a person from whom two superior incisors were extracted on account of decay, and whose places were then filled by two others that had an instant before been taken from a young man; these teeth maintained their places for two or three years, after which the alveolar absorbents consumed these stronger guests, small imposthumes upon the gums followed and the teeth fell out—their roots having been reduced to osseous fillets, which presented a thousand asperities.

Fox has represented a similar case.

I have performed upon myself, and also upon several other persons, an analagous operation, which consists in extracting a diseased tooth, removing its soft central parts, filling it with gold, shortening its extremity, and then replacing it; after five years, this tooth is now as solid as the others;* still, I do not doubt that it will one day end in its being separated from the jaw, for it frequently occasions me very acute local pain, and aggravates the rheumatism to which I am subject. This neuralgia always commences at the tooth and then spreads itself over the whole side of the head; so that I frequently repent having replaced it.

I have extracted teeth that had become very loose, in consequence of inflammation of the periosteum, whose alveolus had acquired a supernatural capacity from the sole action of the absorbing vessels, since there had not been any caries. The periosteum was budlike, very thick, and resembled the fungi-form body that destroys the roots of the primitive teeth. But absorption of this sort is the result of a local disease, determined by the presence of a noisome body in the midst of the parts that it irritates, and which endeavor to dislodge it. This they do, in consequence of the inflammation and pain which give each other mutual support. The first is the instrument, with which life is provided to effect the expulsion, the second calls upon the patient to seek speedier relief.

Nature, in order to open a passage for the molars which are

* It does not enter into the plan of this work to give my sentiments respecting these sorts of engrafted animals. I have tried various experiments with them, which I will describe at another time, and from which I have concluded that the teeth placed in the alveoli, after having been extracted, acquire a sort of vegetable existence by means of a periosteum or false membrane that is developed and holds them in their places.

situated immediately below the gums, and separated from them only by the lamina of bone penetrated by a large orifice, alike accords them the aid of absorption, and the organ charged to perform it, is remarkable for its breadth and thickness. It wears away the gums as well as the maxillary bone, but especially opposite each tubercle of the tooth ; but this body does not possess the redness of those which consume the primitive teeth.

If the absorbents are developed with energy, anteriorly, the the tooth of replacement, that was at first behind, is carried to the side, and the partition which separates it from the temporary is soon destroyed. It is placed immediately below this last, and sometimes ends by pressing beyond it.

Whilst it executes this movement, the posterior lamina of the jaw is gradually brought to meet the anterior, which appears very much to aid the progression of the tooth towards the side. The approach of the two maxillary osseous lamina is very appreciable when we compare the jaws of children of different ages. As soon as a part of the crown of a tooth is engaged in the buccal aperture of the dental matrix, the glutinous liquid, that we have said is therein contained, gradually flows into the mouth. It is probable that when the internal membrane touches the tooth, the contact increases still more the contractile action of this envelope, for the small bone enclosed by it, is then, in a few weeks, raised above the gums, while it has required many years to gain their level.

Although the absorbing apparel be well developed, still, if the *Iter Dentis* is situated too much within the mouth, not only does *the tooth destined to follow its* direction, shoot up without the circle, but also the temporary root either cannot receive any taint, or, as I have before observed, the neighboring temporary will be destroyed, whilst the one that should have been removed remains untouched.

No one, not even the slave of his own laws, can suppose that nature never deviates from the mechanism I have described ; the tooth, it is true, in the greater number of instances, follows the natural route ; but it is not rigorously subject to it. If the absorbing apparel is developed with too much energy on the anterior part, every thing that is found placed before it is rapidly destroyed ; thus the inter-alveolary partition, the root of the temporary tooth, the compact anterior lamina of the jaws, and even the gum that covers it, are alike consumed, so that the point of the tooth, instead of following the canal of the appendage, opens for itself an artificial passage, by piercing either laterally or anteriorly the small matrix. It shows itself very low in the jaw, and below the level of the temporaries, if it is in the inferior jaw; or very high, and above the same teeth, if it is in the superior. These phenomena may from time to time be observed in the bicuspidati, but more frequently still in the conoids. I, for a long time, believed that the deviation of the *Iter Dentis* could alone occasion *gag teeth ;* but anatomical examinations have convinced me that I was in error. Still *gag teeth* are gradually brought into the circle unless some mechanical obstacle opposes them, as, for example, the presence of the temporary teeth, the striking of the anterior teeth, &c. &c. Finally, they are a long while in accomplishing this movement, and it is frequently necessary to determine it by the aid of art.

